# 2023 Southern African HIV Clinicians Society Adult Antiretroviral Therapy Guidelines: What’s new?

**DOI:** 10.4102/sajhivmed.v24i1.1528

**Published:** 2023-09-29

**Authors:** Jeremy Nel, Camilla Wattrus, Regina Osih, Graeme Meintjes

**Affiliations:** 1 Division of Infectious Diseases, Department of Medicine, University of the Witwatersrand, Johannesburg, South Africa; 2 Helen Joseph Hospital, Johannesburg, South Africa; 3 Southern African HIV Clinicians Society, Johannesburg, South Africa; 4 Boston Consulting Group, Johannesburg, South Africa; 5 Division of Infectious Diseases, Department of Medicine, University of Cape Town, Cape Town, South Africa

The Southern African HIV Clinicians Society (SAHCS) has recently published the 2023 update of its adult antiretroviral (ART) guidelines,^1^ continuing the policy of updates every 2–4 years and prompted by changes in the ART therapeutic and research landscape.

## Philosophy

As with previous iterations of the SAHCS Adult ART guidelines, the objective is two-fold. First, to offer best practice guidance for health practitioners working in the Southern African private sector. And, second, to offer guidance for those working in the public sector that complements public sector guidelines with more explanatory detail and advice for common clinical scenarios that are not covered in the public sector guidelines.

A key principle is to harmonise the private and public sector guidelines wherever possible, so that unnecessary differences are eliminated. This is important because it allows for more consistent public health and educational messaging, and because patients may transition between the two sectors as their financial circumstances change. To facilitate this, individuals from the South African National Department of Health ART guidelines committee were part of the 2023 SAHCS ART guidelines committee. There are certain recommendations in the SAHCS Adult ART guidelines that are not included in the South African public sector guidelines for cost reasons (e.g., HIV viral load [VL] test prior to ART initiation) and in these situations, clinicians working in the public sector should follow the public sector guidelines. The guidelines only include treatment and diagnostic options available in southern Africa.

## Tenofovir/lamivudine/dolutegravir-based second-line therapy

Arguably the biggest change in the SAHCS ART guidelines 2023 iteration is the recommendation to recycle tenofovir (TDF) between first- and second-line regimens, instead of switching to zidovudine (AZT), as had previously been recommended.

This guidance was derived from a group of trials published in short succession. The NADIA trial was a randomised trial that enrolled patients who were failing a first-line regimen consisting of tenofovir, lamivudine (3TC) or emtricitabine, and a non-nucleoside reverse transcriptase inhibitor (NNRTI). NADIA randomised participants in a two-by-two factorial manner to (1) dolutegravir (DTG) or darunavir/ritonavir (DRV/r) and (2) switching TDF to AZT, or continuing TDF for the nucleoside reverse transcriptase inhibitor (NRTI) backbone, in each case with 3TC.^2^ The results showed no difference between DTG and DRV/r, with excellent viral suppression rates of > 90% for both options at 48 weeks. Unexpectedly, the strategy of recycling TDF was shown to be non-inferior at 48 weeks, and superior by 96 weeks (92% viral suppression rate vs 85%; 95% CI for difference of 1.2% – 12.8%).^3^ This benefit of recycling TDF was seen even in patients with confirmed high-level resistance to TDF at baseline. The reasons for this finding are likely to be a combination of improved adherence due to a better tolerated once-daily regimen (as opposed to having to take less well-tolerated AZT 12-hourly), and the TDF K65R mutation’s residual ‘crippling’ effect on HIV viral replication.

The favourable findings in the NADIA trial with TDF recycling results were quickly supported by results from VISEND, a large randomised controlled trial conducted in Zambia, and ARTIST, a trial of patients in Cape Town.^4,5^ In the VISEND trial, participants on second-line tenofovir/lamivudine/dolutegravir (TLD) had a superior virological suppression rate at both < 1000 copies/mL and < 50 copies/mL VL thresholds compared to a protease inhibitor (PI)-based regimen that used AZT. In stages 1 and 2 of the ARTIST trial, 82% – 86% of patients on second-line TLD had a VL < 50 copies/mL at 24 weeks.

Put together, these trial findings support TLD as the second-line ART regimen of choice for those who fail an NNRTI-based first-line regimen. In this setting, TLD offers a more tolerable, once-daily, cheaper, and more scalable second-line regimen than previous AZT-based regimens. A further implication is that the switch from NNRTI-based therapy to TLD can now be made, regardless of the patient’s VL, since both 3TC/FTC and TDF can be recycled with DTG, whether or not there is resistance to these drugs.

Thus, most people living with HIV can be expected to be on TLD, given that it is both the first-line therapy of choice as well as the recommended second-line therapy for those who have failed NNRTI-based first-line ART. Certain patients requiring third-line therapy after failing a second-line PI regimen may also be eligible for TLD. There are only limited exceptions to the recommendations for TLD, namely:

- Patients for whom TDF is contraindicated (e.g., chronic kidney disease or severe osteoporosis), in whom abacavir (ABC) or tenofovir alafenamide (TAF) should be considered.
- Patients who do not tolerate DTG, where efavirenz or rilpivirine (RPV) are alternatives.
- Rare patients who develop pure red cell aplasia on 3TC, who are. These patients can be switched to a dual regimen such as TDF + DTG (or RPV + DTG, provided the VL is suppressed).
- Patients who develop resistance to DTG. In these cases, DRV/r can be used instead, provided that DTG resistance is proven by resistance testing first.
- Patients who have developed PI-resistance on LPV/r or ATV/r-based second-line regimens and have cross-resistance to DRV/r. The guidelines recommend against prescribing TLD for such patients, but rather using TLD plus DRV/r unless there is high-level resistance to DRV/r.

## Abacavir in second-line therapy

The evidence for recycling TDF between first- and second-line ART is based on clinical trial data, but the same clinical trial data do not exist for recycling ABC, and it is not known whether the TDF evidence can be extrapolated to ABC. Virologically, the most common mutation seen with ABC is L74V rather than K65R, and there is evidence that the former causes a similar decrease in viral replication fitness to the latter.^6^ Abacavir-based regimens also offer potential adherence benefits since they can be given once-daily and are well tolerated. For these reasons, the guidelines recommend recycling ABC across first- and second-line ART for those patients in whom TDF is contraindicated, despite the lack of clinical trial data.

The implication of both TDF and ABC being recycled across regimens is that AZT therefore no longer forms part of any routinely recommended regimen – a welcome change given AZT’s propensity to cause mitochondrial toxicity.

## Tenofovir alafenamide

For the first time, TAF is included as an option in the guidelines. This is an alternative tenofovir prodrug to TDF, with an improved renal and bone side-effect profile.^7^ When used alone, TAF can be used down to an estimated glomerular filtration rate (eGFR) of 15 mL/min per m^2^, as well as in renal dialysis patients. However, in South Africa it is frequently co-formulated with emtricitabine, in which case its use is limited to patients with a minimum e GFR of 30 mL/min per m^2^. Thus the 2023 SAHCS guidelines recommend TAF as the drug of choice in patients with chronic hepatitis B, and either an e GFR of 30 mL/min per m^2^ – 50 mL/min per m^2^ or osteoporosis. It can also be used as an alternative to ABC in this e GFR range in patients without either of these two comorbidities. However, TAF has some downsides that the SAHCS guidelines committee felt precluded a broader frontline recommendation: compared to TDF it is associated with greater weight gain, an adverse lipid profile, and several important drug-drug interactions.

## Protease inhibitors

Although the 2020 SAHCS guidelines laid out a clear order of preference for the choice of PIs – darunavir as first choice, atazanavir as second, and lopinavir third – the 2023 guidelines are more emphatic in recommending against lopinavir in almost all circumstances. Several studies have shown significantly better viral suppression rates with darunavir compared to lopinavir, likely driven by lopinavir’s poor gastrointestinal side-effect profile and the need to take the drug twice daily.^8,9,10,11^ Lopinavir is also associated with a worse lipid profile compared to other PIs. Therefore, the sole recommendation for use for lopinavir is when a patient requires both a PI and rifampicin-based tuberculosis therapy, because double-dosed lopinavir/ritonavir remains the only PI that can used in this situation. In practice, this scenario is rare since DTG-based regimens can be used in second-line therapy in almost all scenarios.

## How to manage creatinine changes associated with starting dolutegravir

**FIGURE 1:**
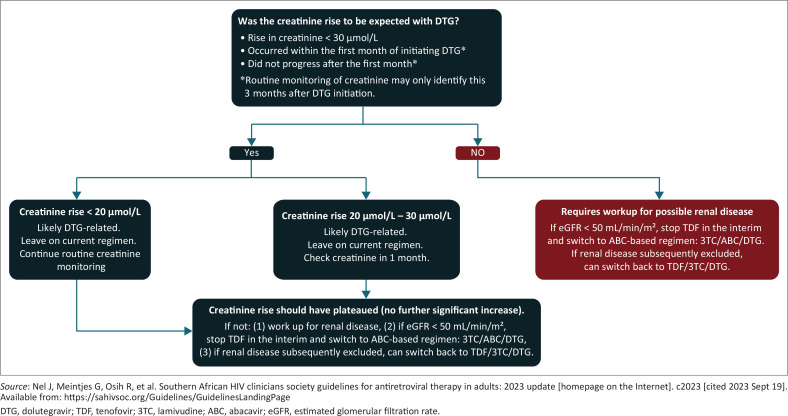
Management of creatinine rise in patients on dolutegravir.

A rise in creatinine when patients are commenced on a DTG-based regimen is a common and vexing issue for clinicians. Dolutegravir inhibits organic cation transporter-2 (OCT-2), thereby inhibiting creatinine’s renal secretion. A rise in serum creatinine concentrations is therefore frequently seen with commencement of the drug. Serum creatinine typically rises within the first few days of starting the DTG, and plateaus after 1–2 weeks. Crucially, it does not represent renal damage and, if the drug is stopped, creatinine levels fall rapidly to baseline again. However, since DTG is typically co-administered with TDF, the clinician is faced with the unwelcome task of trying to distinguish benign DTG-induced creatinine rises with the much rarer but more sinister renal impairment associated with TDF. The 2023 SAHCS guidelines offer a pragmatic approach, based on the pattern of DTG rise observed in the South African-based ADVANCE trial (see Figure 1).

## Other changes

The 2023 SAHCS guidelines contain several other substantive updates, including guidance on enhanced screening for tuberculosis and sexually transmitted infections, updated guidance on tuberculosis preventative therapy (now including options for rifapentine-based regimens), expansion of the module on HIV and mental health, and updates to the guidance on ART and HIV monitoring during pregnancy, amongst others. These are summarised in Table 1. The 2023 Adult ART guidelines thus continue the SAHCS’s tradition of providing high-quality, evidence-based guidance, focusing on practical advice for clinicians.

**TABLE 1:** Selected key updates in the 2023 Southern African HIV Clinicians Society Adult Antiretroviral Therapy Guidelines.

Update	Comment
Recycling of tenofovir in second-line regimens	Evidence from the NADIA and VISEND trials suggest better outcomes than when TDF was switched to AZT as had been previously recommended.
Tenofovir alafenamide	TAF can be considered when patient’s eGFR is 30 mL/min per m^2^ – 50 mL/min per m^2^ or the patient has osteoporosis, especially when they have chronic hepatitis B coinfection.
Patients who return to care after stopping ART	Majority of patients can be re-initiated on TLD, unless there is a reason not to use one of these drugs or the patient requires a more robust third-line regimen.
Choice of protease inhibitor	DRV favoured, with ATV as an alternative. Guidelines recommend against LPV unless there is no other available protease inhibitor option.
Managing creatinine rises associated with dolutegravir	Practical guidance offered based on the pattern and degree of the creatinine rise.
Expansion of tuberculosis screening	Sputum NAAT test (like Xpert MTB/RIF Ultra) should be considered even in the absence of symptoms. Criteria for urine LAM testing expanded.
Inclusion of screening for STIs at baseline	Syphilis serology and NAAT test for *Chlamydia trachomatis* and *Neisseria gonorrhoea* even in the absence of symptoms.

TDF, tenofovir; AZT, zidovudine; TAF, tenofovir alafenamide; eGFR, estimated glomerular filtration rate; ART, antiretroviral treatment; DRV, darunavir; ATV, atazanavir; LPV, lopinavir; NAAT, nucleic acid amplification test; STIs, sexually transmitted infections; TLD, tenofovir/lamivudine/dolutegravir; LAM, lipoarabinomannan.
